# Spatial Point Pattern Analysis of Human Settlements and Geographical Associations in Eastern Coastal China — A Case Study 

**DOI:** 10.3390/ijerph110302818

**Published:** 2014-03-10

**Authors:** Zhonghao Zhang, Rui Xiao, Ashton Shortridge, Jiaping Wu

**Affiliations:** 1 College of Environmental and Resource Sciences, Zhejiang University, Hangzhou 310058, China; E-Mails: zzh8705@163.com (Z.Z.); xr_2003@163.com (R.X.); 2 Department of Geography, Michigan State University, East Lansing, MI 48823, USA; E-Mail: ashton@msu.edu; 3 Ocean College, Zhejiang University, Hangzhou 310058, China

**Keywords:** Ripley’s K function, point pattern analysis, human settlements, geographical associations, Geographic Information Systems, southeastern coastal China

## Abstract

Understanding the spatial point pattern of human settlements and their geographical associations are important for understanding the drivers of land use and land cover change and the relationship between environmental and ecological processes on one hand and cultures and lifestyles on the other. In this study, a Geographic Information System (GIS) approach, Ripley’s K function and Monte Carlo simulation were used to investigate human settlement point patterns. Remotely sensed tools and regression models were employed to identify the effects of geographical determinants on settlement locations in the Wen-Tai region of eastern coastal China. Results indicated that human settlements displayed regular-random-cluster patterns from small to big scale. Most settlements located on the coastal plain presented either regular or random patterns, while those in hilly areas exhibited a clustered pattern. Moreover, clustered settlements were preferentially located at higher elevations with steeper slopes and south facing aspects than random or regular settlements. Regression showed that influences of topographic factors (elevation, slope and aspect) on settlement locations were stronger across hilly regions. This study demonstrated a new approach to analyzing the spatial patterns of human settlements from a wide geographical prospective. We argue that the spatial point patterns of settlements, in addition to the characteristics of human settlements, such as area, density and shape, should be taken into consideration in the future, and land planners and decision makers should pay more attention to city planning and management. Conceptual and methodological bridges linking settlement patterns to regional and site-specific geographical characteristics will be a key to human settlement studies and planning.

## 1. Introduction

The global population exhibits great spatial variability both in settlement patterns and in impact on Earth’s ecosystems [1]. Settlements provide a spatial focus for most human activity, and therefore also strongly affect local land cover, water quality, and biodiversity [2]. Consequently, human settlement acts as the most fundamental link between people and Earth, and reflects the interaction of people with the surrounding environment [3,4]. Settlement locations are determined by local amenities, economic factors, communications [5,6], and are always subject to food availability and production capacity [7,8]. They are thus influenced to a large extent by topography, water accessibility, and transportation proximity [9,10,11]. As a result, the locations of human settlements are unevenly distributed across various spatial scales. Analyzing the spatial patterns of settlements can contribute to greater understanding of land use changes, ecological processes, cultures and lifestyles, *etc.*

Many recent case studies have been conducted to analyze the spatial patterns of human settlements. Previous studies focused on the area, density and shape of human settlements [12,13]. However, the spatial characteristics of settlement location itself received little attention. The locations of human settlements can be simplified as multiple points within a given space and scale. Fewer studies have characterized the spatial point patterns of settlement locations [14,15,16]. 

Many approaches were used to explore the spatial point pattern of human settlement, such as nearest neighbor distance technique and hazard functions [14,16,17]. Nearest neighbor distance technique was widely used in many previous studies, due to its simplicity and ease of implementation [18,19]. Ripley’s K function is another classical spatial point analysis method, which can extract the spatial characteristics of point data from digital images [20,21,22,23]. This function is a second-order statistical method which is based on the distribution of the distances of points and is able to describe fine- and medium-scale spatial correlation pattern of these points. It has been widely used in wildlife habitat characterization [24,25,26,27,28,29], but it may also offer useful insight about the spatial characteristics of human settlements. As the statistic investigates the degree of spatial association across multiple spatial scales, Ripley’s K function could provide more information on the scale of the pattern than nearest neighbor distance metrics [30,31]. We maintain that the scientists and decision-makers can determine the current spatial patterns of human settlement with this function, and that this information may make the consequences of the plans and policies clearer. Understanding the size, pattern and spatial distribution of human settlements is fundamental for distributing resources, settlement management and socio-economic development [17,32,33]. Moreover, unplanned distribution of human settlement can impair ecosystems’ capacities to deliver services and raised various environmental problems, such as polluted soils and rivers, disrupted water cycles, decreased species diversity, and increasing public health risks [34,35].

Site- and situation-specific geographical factors can critically influence human settlement patterns. Liu *et al.* [12] examined the topographical factors of human settlement expansion and observed that the lands with lower gradient were always more favorable for development. Li *et al.* [36] explored geographical differences in spatial pattern of human settlements environmental suitability in the Three Gorges Reservoir Area in central China and concluded that the human settlements distribution is closely related to the terrain. Wei *et al.* [37] studied the spatial pattern of human settlements in the Shiyang River Basin and indicated that spatial distribution of population is profoundly affected by severe environmental constraints, such as the expanding deserts, hilly terrain and the changing climate, and concluded that some residential areas should be relocated. Similarly, we proposed that development patterns of different counties in Wen-tai region, in eastern coastal China, have been differentially influenced by their particular geographical factors. The key question in recent years facing land planners and decision makers in this region is how to manage human settlement to balance the increase of population and loss of farmland [38]. For the coastal areas, rapid socio-economic development was anticipated to result in dramatic population growth and human settlement expansion. The goal of this study is to investigate the spatial variation in geographical associations or influences on current settlement patterns. Understanding this variation could provide valuable insight for planners and decision-makers as they respond to and prepare for this growth.

**Figure 1:**
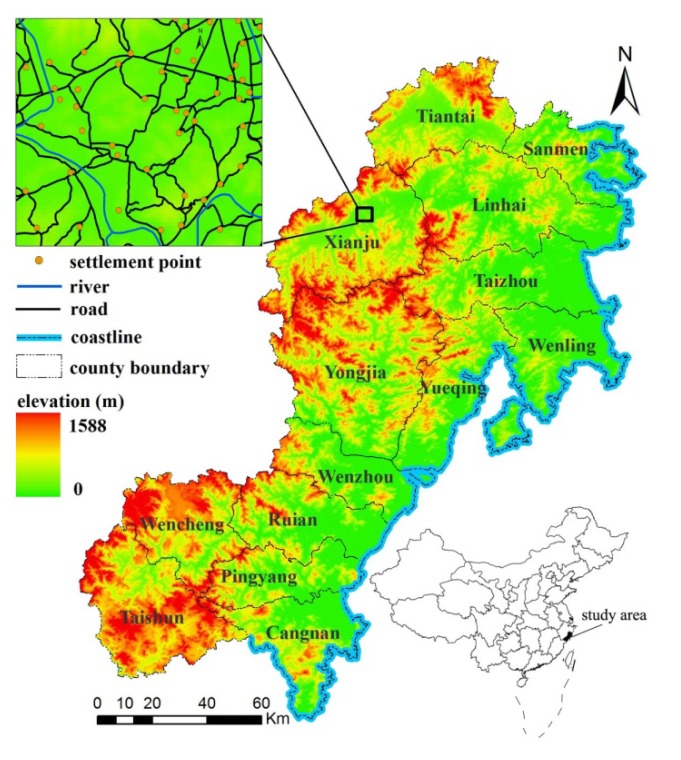
Location of the Wen-Tai region, Zhejiang Province, China.

The Wen-Tai region is located in southeastern coastal China (Figure 1) within the province of Zhejiang, with a spatial extent of 27°03′–29°08′N and 119°37′–121°26′E. It contains 16 counties, covers 21,667 km^2^, and had a population of about 13.7 million in 2008 [39]. This region has a subtropical monsoon climate with moderate temperatures, abundant precipitation, and distinct seasons. The average annual temperature in this region is 18.8 °C, and annual rainfall is 1,922 mm. The topography of this region is rugged: about 75% of the total area is mountainous. The eastern coastal region plain is lower and more level, while the western regions are comprised of valleys surrounded by steep mountains, typically covered in dense forests. Like other parts of eastern coastal China, the Wen-Tai region has recently witnessed explosive socio-economic development. Over the past twenty years the population in Wen-Tai increased by 16%, and GDP increased by 3,475% [39]. A unique aspect of Wen-Tai is its distinctive economic development patterns. From the 1980s, the Chinese government implemented a series of policies such as “the market transition” to promote an export-oriented economy in eastern coastal cities [40]. Such policies, coupled with the terrestrial inaccessibility of Wen-tai, resulted in the “Wenzhou model”, in which local private business developed rapidly, facilitated by provincial and especially municipal government [41]. Wen-Tai relies heavily on its own resources, and local governance, and is therefore less affected by national trends and policies than other regions in coastal China. As a consequence, settlement locations within this region are mainly affected by local and geographical factors. Wen-Tai provides a useful case study for the analysis of geographical associations with settlement patterns.

This paper aims to apply spatial point pattern analysis to investigate human settlement patterns and their geographic influences across the Wen-Tai region. Our objectives are to (1) evaluate the capability of Ripley’s K in exploring the spatial point pattern of human settlements; (2) analyze the local variations of settlement point pattern across this region; and (3) interpret geographical associations of settlement spatial point patterns.

## 2. Materials and Methods

### 2.1. Data Collection

The settlement data (1: 50,000 scale; year 2008) were obtained from the Zhejiang Province Surveying and Mapping Bureau and digitized in ArcGIS 9.2 Desktop (ESRI Inc., Redlands, CA, USA) software. The centroid coordinate of each administrative village (with approximately 500 persons on average in Wen-Tai region) was used to represent its location for all villages recorded in the field survey; the inset in (Figure 1) shows a representative local area with settlements.

Digital maps (1:50,000) of county road/river networks and coastline were also provided by the Zhejiang Province Surveying and Mapping Bureau. Surface elevation, slope, and aspect were derived from 30m spatial resolution ASTER GDEM (version 2) [42].

The Euclidean distance output rasters record the straight-line distance from the center of every cell to each nearest source (road/river networks or coast line), such as meters are computed from cell center to cell center. The Euclidean Distance function in ArcGIS software is used frequently as an effective function for similar applications. If *q* is the nearest point on a geographic element of interest (e.g., a road/river network or coast line) for settlement point *p*, and the location of *p* and *q* are *(p_1_, p_2_)* and *(q_1_, q_2_)*, then the distance *D*_(p,q)_ is given by:

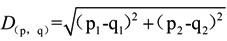
(1)

### 2.2. Spatial Point Pattern Analysis

The behavior of a general spatial distribution process can be characterized in terms of its first-order and second-order properties. First-order properties describe the spatially varying intensity of a point pattern, in which intensity is defined as the expected (mean) value of the distribution at locations throughout the region of interest [30]. Second-order properties describe the covariance (or autocorrelation) structure of the point pattern and can be identified by analyzing the distribution of distances between those sample points [15,43,44].

Ripley’s K function is regarded as a suitable tool to characterize second-order properties of a point pattern [45]. It is the expected number of points in a circle of radius *d* with a random point at center, and is formally defined as:

*K(d) = λ^ −1 ^E(f)* (2)

where *E(f)* is the expected number of other sample points within distance *d* of a sample random point and *λ* is the intensity of sample points per unit area. Since the expected number of sample points within a distance *d* of a chosen random point in a process with no spatial dependence is *λπd^2^*, *K(d)* for a spatially random process can be defined as [30,46]:

*K (d) = πd^2^* (3)

If the points display a clustered pattern, an excess of sample points at short distances can be shown. The empirical function is defined as:

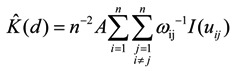
(4)
where *n* is the number of events in the analyzed plot, *A* is the area of the plot (m^2^), *I* is a counter variable, *u_ij_* is the distance between events *i* and *j*, and *w_ij_* is a weighting factor to correct for edge effects. In our study, toroidal edge correction was used to avoid the edge effects by treating the rectangular study plot encompassing the study region as a torus, that is, the part of a sample outside the rectangle is made to appear at the corresponding opposite border [47]. Points at opposite sides of the plot are now close to each other and the boundary does not exist [48].

The Monte Carlo technique was employed to test CSR through Ripley’s K function with 95% significance level. Monte Carlo simulation is useful to provide empirical models of uncertainty by which the statistical significance of results can be quantified [49]. Much of the spatial point pattern is undertaken by Monte Carlo simulation because of the complexity of spatial point processes [50,51]. There are three broad classes of second order point processes. The first is called complete spatial randomness (CSR) [52,53,54], in which points are independent of each other and the objects are distributed as a result of random chance. The second is called a cluster process, in which points are associated with each other and objects follow a certain process of concentration. The third is a regular process, in which points are generally separated from one another with some spatial inhibition. The Monte Carlo approach is used to simulate randomly generated point patterns and it is assumed that the random number generator employed is satisfactory [52]. The simulated point map is then analyzed like the original pattern. This procedure is repeated 20 times and the lowest and highest value of *K(d)* for each *d* is used to define the lower and upper bound of a 95% confidence envelope for a CSR process. If the plot of *K(d)* for the original pattern falls between the lower and upper bounds, the observed spatial structure can not be statistically distinguished from a CSR process. Otherwise, the structure of the original pattern may be determined to be significantly more clustered or regular than random. The Programita software [43] was used in this study to calculate Ripley’s K function and Monte Carlo simulation. 

### 2.3. Statistical Analyses

**Figure 2:**
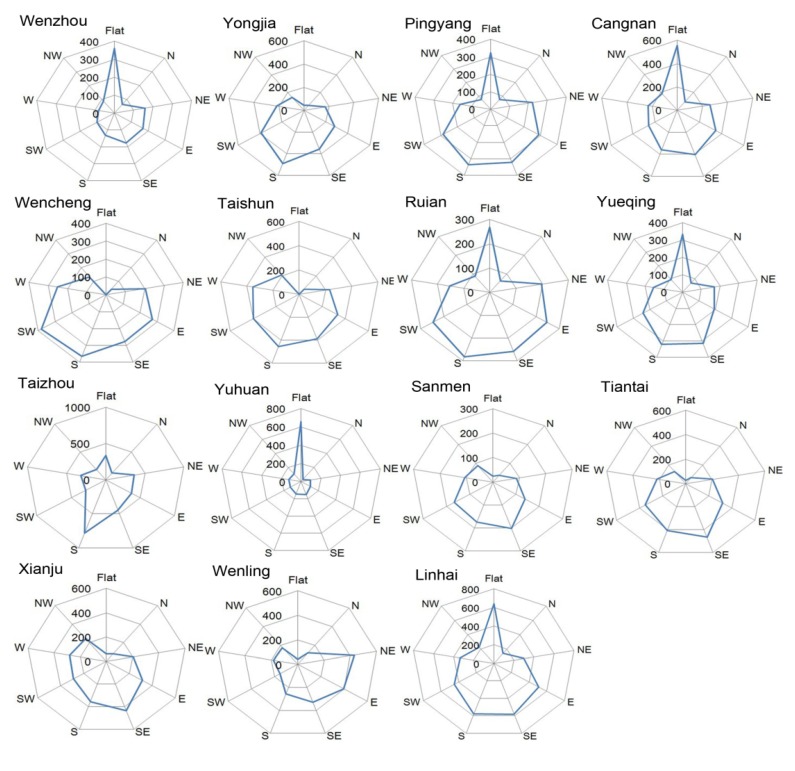
Rose plots of the human settlement aspect in Wen-Tai region, China (N: North; NE: Northeast; E: East; SE: Southeast; S: South; SW: Southwest; W: West; NW: Northwest).

Stepwise multiple linear regression was performed to analyze the geographical determinants of settlement spatial point patterns. We chose the human settlements patterns as the dependent variables. Ripley’s K function classified the spatial point patterns into three categories: regular, random and cluster. We assigned regular pattern as 1, random pattern as 2 and cluster pattern as 3. Independent variables included mean and standard deviation value of elevation (meters), slope (degree), distance to river (meters), distance to road (meters), distance to coast (meters) and aspect (degree). These variables were selected because they were evidenced to play important role in determining human settlement patterns [10,55,56,57,58]. The ArcGIS 9.2 Spatial Analyst tool [59] was used to calculate corresponding values of the selected variables for each point. All regression models were performed through SPSS 16.0, after all data were standardized and normalized. General descriptions of geographical characteristics were shown in Table 1 and Figure 2.

**Table 1:** Mean and standard deviation (STD) of settlement distance from road/river networks and topography of each county in Wen-Tai region, China.

Study Area	N	Road Distance		River Distance		Coast Distance		Elevation		Slope	
		Mean	STD	Mean	STD	Mean	STD	Mean	STD	Mean	STD
Wenzhou	1,330	102.26	107.08	194.52	182.49	22,758.62	9,940.18	133.79	189.53	4.61	4.92
Yongjia	2,152	129.93	144.47	244.96	208.77	19,275.30	10,088.43	324.11	229.97	9.47	4.74
Pingyang	2,096	139.73	126.39	187.21	157.58	56,582.07	9,748.80	179.67	183.60	6.84	5.37
Cangnan	2,699	109.7	118.21	164.31	157.51	12,291.24	10,367.18	159.38	154.98	5.78	4.80
Wencheng	1,925	104.68	102.12	216.98	162.82	71,509.33	10,617.83	497.50	221.11	9.73	5.03
Taishun	2,433	110.47	109.15	178.61	139.42	65,038.85	13,592.32	547.19	175.01	8.49	4.41
Ruian	1,805	104.52	107.19	182.51	147.61	35,348.65	17,795.03	186.81	195.42	7.11	5.41
Yueqing	1,891	143.00	136.51	207.54	181.35	30,343.00	12,869.21	131.61	152.25	5.45	4.80
Taizhou	3,544	88.27	102.25	218.85	200.29	9,059.92	6,216.39	89.48	154.90	3.52	5.01
Yuhuan	905	92.15	114.83	219.19	179.66	23,361.77	13,223.02	67.88	67.53	4.52	2.99
Sanmen	1,025	38.81	60.88	182.66	167.45	2,051.22	1,603.43	83.89	99.58	5.17	3.45
Tiantai	2,217	42.93	62.44	193.21	203.82	7,487.47	5,144.85	268.94	206.49	5.58	4.43
Xianju	2,242	55.16	86.40	220.87	210.66	64,487.73	9,985.80	305.10	220.50	7.96	5.34
Wenling	2,688	78.05	88.28	164.12	153.96	35,348.65	17,795.03	41.61	56.64	2.50	3.41
Linhai	3,371	54.15	109.54	194.84	192.32	6,856.95	4,583.17	143.62	165.77	5.63	4.80

Note: N is the number of human settlement points.

## 3. Results and Discussion

### 3.1. Spatial Point Pattern of Human Settlements

**Figure 3:**
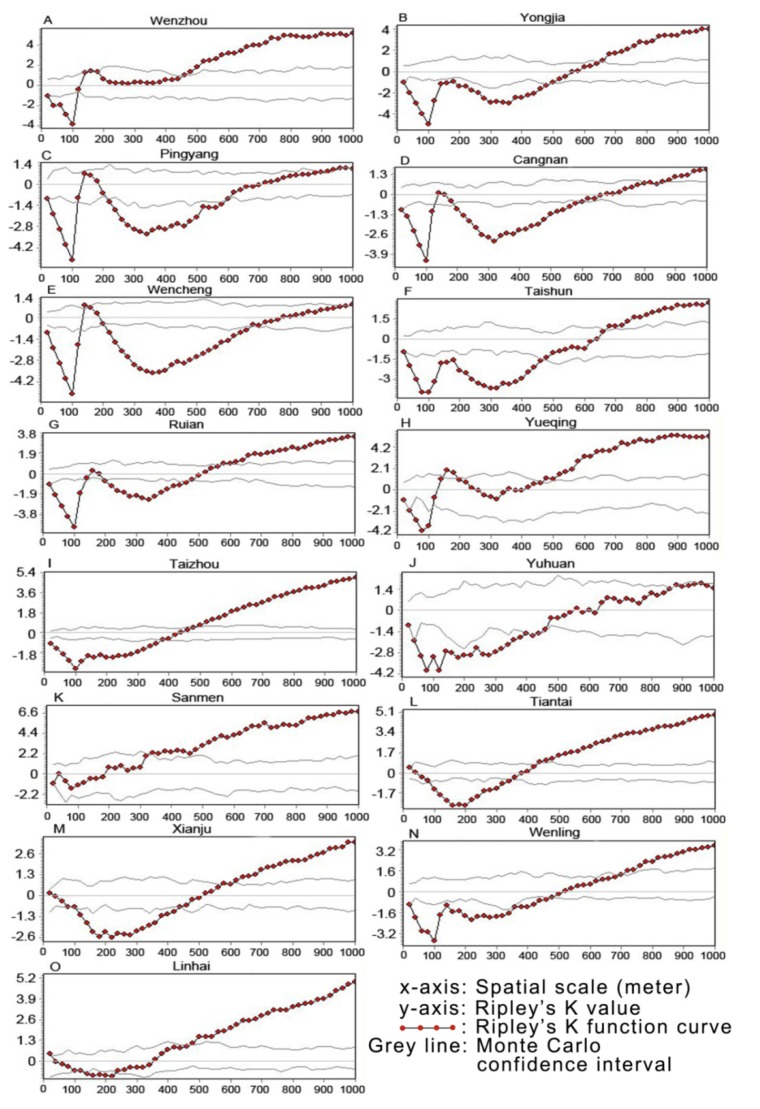
Ripley’s K point pattern of human settlements in Wen-Tai region, China. Figures show the Ripley’s K function curve (grey line with red dots) between the two grey lines as the Monte Carlo confidence interval in different counties.

Monte Carlo methods were used in this study to create a CSR confidence envelope for the Ripley’s K for these spatial point patterns. Results showed quite variable confidence envelope width and shapes across spatial scales for different counties (dotted lines in Figure 3). Relatively smooth envelopes were generated for counties with large numbers of settlements (e.g., Taizhou and Tiantai), while rougher envelopes were generated in counties with fewer numbers of settlements (e.g., Pingyang, Wencheng and Ruian). The Monte Carlo approach was conceptually simpler and more generally applicable than analytical derivations, but required substantial computational resources because it relied on simulation to generate critical values for significance testing [30,52].

Results of Ripley’s K function analysis are shown in Figure 3. The x-axis on each plot reports *d*, or distance in meters, while the y-axis is a transformed measure of K such that positive values are indicative of clustering and negative values are indicative of regularity. It indicated that settlement point patterns throughout Wen-Tai followed a consistent pattern across geographic scales. 

At the finest scale (0–200 m.), settlements tended to be regularly distributed, as represented by negative values of *K(d)* at those distances. At medium scales, *K(d)* tended to fall within the envelopes, indicating no significant departure from randomness. At longer scales, *K(d)* tended to lie above the envelopes, indicating clustering. However, there were interesting departures from this pattern. Specifically, Yueqing, Tiantai and Xianju had similar settlement point distribution patterns, representing random → regular → random → cluster with scale increases, with increases above the envelope at 500 m (Yueqing and Tiantai) and 600 m (Xianju). Other counties such as Pingyang, Cangnan and Wencheng showed regular → random → regular → random → cluster patterns with scale increases. They largely attained a cluster pattern at the 1,000 m scale, indicating that settlement points showed scattered and random distribution at finer spatial scales. Other counties, such as Wenzhou, Yongjia, Ruian, Taizhou and Wenling, observed regular → random → cluster pattern with scale increases, and their K rose above the CSR envelope at the 400–700 m scale. It must be noted that the Ripley’s K function assumes there is no first-order process, indicating the presence of a spatial trend. In the presence of such a trend larger-scale values of K may be inaccurately estimated.

It is worth speculating on why the general pattern is observed throughout the study region. Regularity at the finest scales could be due to several factors: limited precision of measurement of the source data, minimum size of management unit, or minimum land area needed to maintain a settlement. The first is essentially an error component, while the second is related. The third factor is more intriguing: subsistence agricultural settlements require additional land for resource extraction. The consistent minimum distance may reflect the area needed in this part of the world to maintain a settlement. Some county patterns observe clustered peaks at around 200 m, which suggests that in these areas groups of settlements are concentrated at that distance range. Regularity at distances of 300–500 m in some graphs suggests that clusters of settlements are relatively evenly spaced, with patches of open land between the clusters. Finally, the presence of sustained long-range clusters could indicate that substantial areas of each county have denser settlement patterns than other areas—that is, settlement density at the 1 km scale and up is not uniform. Previous researches have emphasized the importance of studying spatial pattern of human settlements. Yang *et al.* [60] found that some different-sized cities had common characteristics of settlement pattern and indicated that policymakers should pay more attention to future city planning and management. Our findings suggest that the spatial association of settlements is complex, depends upon the scale at which association is measured, and varies from place to place, even within a subnational region.

### 3.2. Geographical Associations

**Figure 4:**
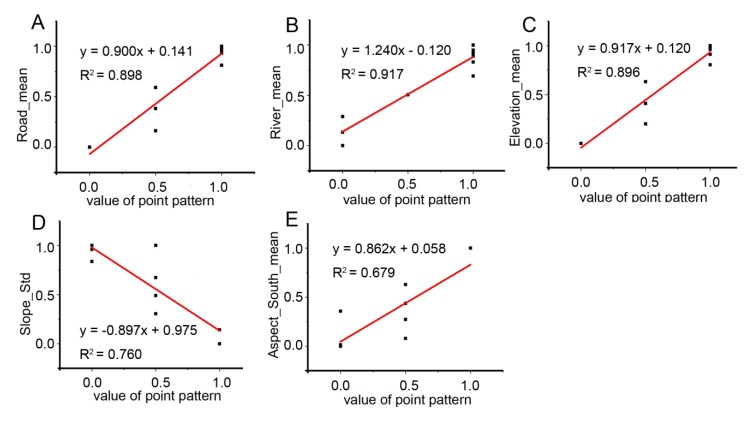
Relationships between different geographical association variables and value of point pattern in different counties: (**A**). Yueqing; (**B**). Tiantai; (**C**). Wenzhou; (**D**). Pingyang; (**E**). Wencheng. (After normalization processing we got 0.0 for regular pattern, 0.5 for random pattern and 1.0 for cluster pattern (lateral axis). For the same processing we got the value of geographical association variables from 0.0 to 1.0 (vertical axis).)

The geographical associations of settlement spatial point patterns are displayed in Table 2. Distance to road was the primary predictor for settlement point patterns in Yongjia, Yueqing (Figure 4A), Taizhou, Yuhuan and Linhai, while the average distance to river was positively correlate in Yongjia, Sanmen and Tiantai (Figure 4B), and standard deviation of distance to coastline was positively correlate in Wenling. Figure 1 showed that Sanmen and Tiantai were located in the northern region, and Table 2 represented that the settlement point patterns were significantly affected by the river networks in these two areas. Such results implied that settlement locations were regular near the river and clustered far from the river in this part. We speculate that settlements near the river were sparser due to the risk of flooding.

In hilly regions the influence of topography was more pronounced. Mean elevation shows positive coefficients for Wenzhou (Figure 4C), Taishun and Wenling, standard deviation of elevation displays positive coefficients for Ruian and Xianju, suggesting that human settlements patterns in those counties are significantly explained by elevation and elevation variation. 

**Table 2:** Point pattern estimated from multiple linear regression models.

Study Area	Multiple Linear Regression Models	R^2^
Wenzhou	0.917 × elevation_mean + 0.12	0.896 ^**^
Yongjia	−0.963 × road_mean + 1.712 × river_mean + 0.348	0.899 ^* ^
Pingyang	−0.897 × slope_std + 0.975	0.760 ^**^
Cangnan	−1.169 × river_std + 1.067	0.910 ^**^
Wencheng	0.862 × aspect_south_mean + 0.058	0.679 ^**^
Taishun	1.143 × elevation _mean − 0.045	0.943 ^**^
Ruian	1.048 × elevation _std + 0.022	0.757 ^**^
Yueqing	0.9 × road_mean + 0.141	0.898 ^**^
Taizhou	1.134 × road_mean − 0.11	0.930 ^**^
Yuhuan	−1.214 × road_std + 1.244	0.750 ^**^
Sanmen	0.783 × river_mean + 0.296	0.797 ^**^
Tiantai	1.24 × river_mean − 0.12	0.917 ^**^
Xianju	1.185 × elevation _std − 0.13	0.919 ^**^
Wenling	1.190 × elevation_mean + 0.443 × coast_std − 0.454	0.924 ^**^
Linhai	−0.806 × road_mean + 0.521 × slope_mean + 0.785	0.886 ^* ^

Notes: ^**^ Significant at the 99% confidence level. ^*^ Significant at the 95% confidence level. Abbreviation: Mean/standard deviation value of elevation (elevation_mean/elevation_std), Mean/standard deviation value of slope (slope_mean/slope_std), Mean/standard deviation value of distance to river (river_mean/river_std), Mean/standard deviation value of distance to road (road_mean/road_std), Mean/standard deviation value of distance to coastline (coast_mean/coast_std) and Mean/standard deviation value of aspect (aspect_south_mean /aspect_south_std, aspect_southeast_mean / aspect_southeast_std *et al.*).

Standard deviation of slope exhibited negative coefficients for Pingyang (Figure 4D), where there was also a substantial amound of *K(d)* values falling within the CSR envelope (Figure 3). This may be because Pingyang includes a coastal area in the east and a rugged western area in the interior. Therefore slope exerted more significant impact on settlement point patterns in this county.

Aspect factor showed positive coefficients for Wencheng (Figure 4E). Moreover, aspect graphs (Figure 2) exhibited that this county mainly had south and southwest aspect without many level sites. It indicated that settlements in Wencheng facing south and southwest aspect presented regular patterns. In addition, the positive correlation suggested that residents living on the hills far from the sea had to be selective about appropriate facing slopes, perhaps to maximize seasonal insolation or due to associated factors like soil quality. Across most spatial scales settlement patterns appeared to be regular or random in this county. We note that in southern China people prefer to build their houses facing south. In summer, less sunlight can get in when sun elevation is high, while in winter, when sun elevation is low, more sunlight can get into houses through windows, warming them. Topographic aspect may be associated with this.

### 3.3. Implications for Regional Human Settlement Planning

Human settlement areas are special locations for living and engaging in economic, political, and cultural activities, and are fundamental local-scale drivers of land use and land cover. Since the absence of uniform planning or strong execution for human settlements in old times, the spatial distribution of human settlement is characterized with separated and weak aggregated level. This unplanned human settlement distribution can bring severe waste of land resources and irrational built-up land structure, and restrict the processes of industrialization, urbanization and modernization in rural areas. Especially for Wen-Tai region, which is surrounded by mountains to the west and by sea to the east, the proper land planning in hilly regions is needed to prevent debris flow, landslides and other geological disasters, while coastal areas are prone to suffering hurricanes, tsunamis, *etc.* Settlement locations are affected by geographic factors at the site level, as well as with respect to their regional situation that can promote or inhibit the success of a settlement. In China, ongoing debate has concerned the relative role of large, medium-sized and small settlements in accommodating future urban growth and development. During the 2000s, the Chinese government has promoted many policies that aim to seek a balanced and coordinated development between urban and rural areas. China's special national conditions, including the contradictions between the support of China’s huge population, the environmental carrying capacity of the land, and the economic increase require us to pay more attention to scientific settlement planning. Therefore, land plans and policies should be considered along with local conditions to adjust the regional structure gradually in the future.

In this study, the general distribution characteristics of human settlements across a wide range of spatial scales throughout Wen-Tai were identified through Ripley’s K function; substantial general properties of the spatial structure of settlements was observed, with settlements being regularly distributed at the finest spatial scales and clustered or random at broader scales. However, variation was readily apparent from this general pattern in many counties. These results can provide reference and information for land planners and decision-makers to establish infrastructure and build housing, which is essential for the sustainability of human settlement development. We have demonstrated that associations between geographic factors and settlement patterns differ in different parts of Wen-Tai, and highlighted substantial variations of geographical factors in settlement distributions. 

### 3.4. Limitations and Prospects

Our study used Ripley’s K function and the Monte Carlo method to describe the characteristics of settlement point patterns and formally test them against a CSR hypothesis. Further, we employed regression to identify the effects of topographic factors on the spatial structure of settlement patterns. However, we note several limitations to the study and the approach. Firstly, the results presented here are constrained by the spatiotemporal scales of the data, we investigated the geographical determinants with human settlements patterns only at regional scale and in one year. The study is ahistorical, in that settlement development over time is not considered. Although multi-scale analysis using *K(d)* was conducted, these metrics were only summarized within one spatial framework: counties. Other spatial units (e.g., local scale, watershed scale, administrative scale), and temporal variations between different point patterns of human settlement and the determinants are thus not discussed. Further study will be conducted in this aspect when data will be available. Secondly, we only considered the influence of topographic factors in this study, because the topography of Wen-Tai region is complicated, characterized by upland areas and mid-low hills, which would give significant effects to the distribution pattern of human settlement. Other determinants (e.g., soil, geologic, land use and climate variables) may be usefully considered in the future, as can social variables such as distance to high value crops, distance to natural amenities, hazards, *etc.* Lastly, this study only considered approaches for the measurement and communication of current conditions of settlement patterns. Future study building on these findings to develop models for selecting optimal settlement locations, could contribute to sustainable development in China.

## 4. Conclusions

This study applied Ripley’s K function to analyze human settlement patterns across the Wen-Tai region, China. It was found that settlements across Wen-Tai region generally followed regular → random → cluster from fine to coarse geographic scales. Settlement patterns located on the coastal plain presented more regular and random patterns, while those in interior, hilly areas exhibited clustered patterns. Moreover, areas with clustered settlements were typified by higher elevations with steeper slopes and south facing aspects than were areas with random or regular settlements. Regression showed that effects of topographic factors (elevation, slope and aspect) on settlement locations were stronger in hilly regions. Our study showed that Ripley’s K function effectively characterized the complex spatial structure of human settlements in eastern coastal China, and that Monte Carlo simulation was useful to determine the statistical significance of a particular point pattern analysis.

Different counties have different development models. As a result, urban planners may design different settlement patterns in different contexts. Ripley’s K function can provide quantitative descriptions to inform planners of the settlement patterns. For some counties the distribution of human settlements is random and unplanned at a wide range of scales, which brings disadvantages for built-up planning. After realizing the unplanned distribution patterns of human settlement, land planners and policy makers can use these patterns to evaluate local conditions and responsibly adjust the regional structure in the future. In addition, planners can simply rely on the identified geographical determinants associations to indicate settlement patterns. We therefore advocate the application of Ripley’s K function in urban planning practice. Given the useful information from point analysis, we suggest that the structure of spatial point patterns of settlement locations should be taken into consideration in the future.
